# Kaposi sarcoma in solid organ transplant recipients: updates in epidemiology, diagnosis, treatment and prevention

**DOI:** 10.3389/fimmu.2026.1698179

**Published:** 2026-03-24

**Authors:** Alessandra Mularoni, Andrea Cona, Carlotta Piazza, Francesca Pecoraro, Patrizia Barozzi, Mario Luppi

**Affiliations:** 1 Department of Infectious Diseases and Antimicrobial Stewardship, Mediterranean Institute for Transplantation and Advanced Specialized Therapies (IRCCS ISMETT), Palermo, Italy; 2 Department of Infectious Diseases and Antimicrobial Stewardship, UPMC Italy, Palermo, Italy; 3 Department of Research, Mediterranean Institute for Transplantation and Advanced Specialized Therapies (ISMETT IRCCS), Palermo, Italy; 4 Section of Hematology, Department of Medical and Surgical Sciences, University of Modena and Reggio Emilia, AOU Modena, Modena, Italy

**Keywords:** HHV-8/KSHV seroprevalence, HHV-8/KSHV-associated disease, human herpes virus 8/Kaposi’s sarcoma-associated herpesvirus, Kaposi sarcoma, solid organ transplantation

## Abstract

Kaposi’s sarcoma (KS) is one of the most frequent malignancies observed in solid organ transplant (SOT) recipients, and it is associated with human herpes virus 8/Kaposi’s sarcoma-associated herpesvirus (HHV-8/KSHV) infection. The incidence varies according to the prevalence of HHV-8/KSHV in the population, the intensity of the immunosuppression and serological status of organ donor and recipient. Both latent and lytic phases of the HHV-8/KSHV life cycle play crucial role in the pathogenesis, influencing oncogenesis, immune evasion, and inflammasome activation. KS can be the result of reactivation of HHV-8/KSHV latent infection in the immunosuppressed recipient or be the consequence of a primary infection (either donor- or non-donor-derived), the latter possibly associated with a more aggressive clinical course. KS usually presents with cutaneous lesions, however post-transplant KS is characterized by visceral and/or lymph node involvement, frequently in absence of cutaneous lesions, underlying the challenges associated with KS diagnosis in SOT and the need of high clinician suspicion. The mainstay of post-transplant KS management is reduction of immunosuppression, along with conversion to mTOR inhibitors, while in visceral forms chemotherapy with liposomal doxorubicin is usually the first choice. Active surveillance and personalized management strategies, based on risk stratification and multimodal therapeutic approaches, are essential to optimize outcomes in transplant recipients affected with KS.

## 1 Introduction

Solid organ transplantation (SOT) has improved the survival and quality of life of patients with end-stage organ dysfunction. However, the chronic use of immunosuppressive agents to prevent allograft rejection increases the risk of infection and virus-associated neoplasms. Kaposi Sarcoma (KS), a tumor characterized by the proliferation of spindle endothelial cells, abnormal angiogenesis and inflammatory infiltrates, is the most common human herpes virus 8/Kaposi’s sarcoma-associated herpesvirus (HHV-8/KSHV)-associated disease (KAD) among SOT recipients and it was described in 1872 by the Hungarian dermatologist Moritz Kaposi (1). The first case of post-transplant (PT) KS was reported in 1969 in a woman who had received a kidney transplant from a deceased donor (2), but KS remained uncommon until the early 1980s, when its incidence arose dramatically with the AIDS epidemic. At that time, the etiology of KS was unknown, but an infectious cause was suspected. In 1994, Chang and Moore (3) identified in KS lesions from patients with AIDS unique DNA sequences that belonged to a previously unknown herpesvirus marking a significant advancement in the field of virus-driven oncogenesis (4, 5).

The epidemiology, clinical presentation, and optimal treatment of KS in SOT recipients remain poorly characterized, because most of the current knowledge about HHV-8/KSHV and KAD comes from studies conducted in HIV-positive patients.

This knowledge gap is particularly important given the unique immunological setting of transplantation and the increasing size of this at-risk population. The aim of this review, performed using a search strategy in PubMed with predefined keywords until January 2026, is to give an update on current knowledge regarding epidemiology, clinical characteristics, and treatment of KS in the SOT setting, with the goal of informing both clinical practice and future research. The following search terms were used: (“Kaposi sarcoma- associated herpesvirus” OR “KSHV” OR “human herpesvirus 8” OR “HHV8″) AND (“liver transplantation” OR “liver transplant”) AND (“Kaposi sarcoma” OR “Kaposi” OR “KS”); (“Kaposi sarcoma- associated herpesvirus” OR “KSHV” OR “human herpesvirus 8” OR “HHV8″) AND (“kidney transplantation” OR “kidney transplant”) AND (“Kaposi sarcoma” OR “Kaposi” OR “KS”); (“Kaposi sarcoma- associated herpesvirus” OR “KSHV” OR “human herpesvirus 8” OR “HHV8″) AND (“lung transplantation” OR “lung transplant”) AND (“Kaposi sarcoma” OR “Kaposi” OR “KS”); (“Kaposi sarcoma- associated herpesvirus” OR “KSHV” OR “human herpesvirus 8” OR “HHV8″) AND (“heart transplantation” OR “heart transplant”) AND (“Kaposi sarcoma” OR “Kaposi” OR “KS”).

## 2 HHV-8/KSHV

### 2.1 Characteristics, life cycle and pathogenesis

HHV-8/KSHV is a double-stranded DNA virus, belonging to the family of the gamma herpesvirus that infects B cells, endothelial cells, macrophages and monocytes (6, 7).

Beside KS, HHV-8/KSHV can cause other neoplastic diseases including primary effusion lymphoma (PEL), an aggressive B-cell lymphoma that typically presents as malignant effusions in body cavities, and Multicentric Castleman Disease (MCD), an atypical lymphoproliferative disorder characterized by systemic inflammatory symptoms, high HHV-8/KSHV viral load, cytopenia and multifocal lymphadenopathy. In addition, other HHV-8/KSHV-associated lymphoid entities including germinotropic lymphoproliferative disorder (GLPD), HHV-8/KSHV positive diffuse large B-cell lymphoma and extra-cavitary PEL have rarely been described in SOT recipients (8).

Among non-neoplastic KAD, the Kaposi’s Sarcoma-associated Herpesvirus Inflammatory Cytokine Syndrome (KICS) is a potentially life-threatening disease that manifests, similarly to MCD, with fever, respiratory, gastrointestinal and neurologic symptoms, cytopenia, hypoalbuminemia, hepatosplenomegaly, and lymphadenopathy but without the characteristic histopathology of MCD (9–12). Usually, high viral load and elevated IL-6, IL-10, TNFα, IL 17A and IFNα are present (13).

The main route of transmission for HHV-8/KSHV is saliva, but the virus may also be transmitted through sexual intercourse, the use of contaminated needles, blood transfusions, or organ transplantation (14–16). In SOT population, KS may be the result of different mechanisms: primary infection in a seronegative recipient either donor (D+/R-) or non-donor derived (D-/R-) (10). Donor derived infection usually results in disease with earlier onset and more severe clinical presentation, and concomitant KICS or other KAD may also occur. In seropositive recipients (R+), KS can arise as the consequence of reactivation/uncontrolled expansion of latently infected cells, under immune suppression. KS malignant cells, seeded with the graft, can be transmitted and then proliferate in the immunosuppressed recipient (16).

The life cycle alternates between latent and lytic phases. The latent phase is characterized by the persistence of the virus in the nucleus of infected cells, expressing latency genes, such as LANA-1, v-FLIP, and v-Cyclin, and microRNAs that promote cell survival and proliferation, inhibition of apoptosis and vascular proliferation and are therefore implied in the pathogenesis of KS; indeed, in KS the majority of infected cells are in the latent phase (7, 14).

The lytic phase is triggered by expression of the immediate-early transactivator RTA, also known as ORF50, which functions as the latent-to-lytic switch by activating viral and cellular promoters, recruiting epigenetic cofactors, and inducing the early gene cassette required for ori-Lyt–driven DNA replication; this culminates in synthesis of viral DNA and production of mature virions. During this cascade, lytic genes with paracrine activity, including vIL-6, vGPCR and the viral chemokines vMIP-I/vMIP-II, are induced and contribute to systemic inflammation and a proangiogenic microenvironment.

According to this, Lage et al. (17) suggest that inflammation is not merely epiphenomenal but a driver of KS initiation and progression. KS arises where HHV-8/KSHV latency and intermittent lytic reactivation intersect with a proinflammatory, proangiogenic microenvironment. Inflammation thereby sustains viral persistence, promotes lytic bursts, and drive the invasive spindle-cell phenotype characteristic of KS lesions (17–19).

Mechanistically, inflammatory pathways (IL−1, IL−6, TNF/NF−κB, COX−2/arachidonate mediators) are recurrently upregulated in HHV-8/KSHV infection. These axes promote KSHV reactivation and tumor cell invasiveness (e.g., MAP4K4→COX−2/MMPs) and maintain the cytokine-rich milieu that is pathognomonic of KS (20, 21). This provides a biological bridge to HHV−8–driven inflammatory syndromes (MCD, KICS) and to PT complications, where iatrogenic immunosuppression permits high level viremia and cytokine excess, correlating with, cytopenia, and KS flares; HHV-8/KSHV–linked bone marrow failure exemplifies how systemic inflammation and lytic activity extend pathogenesis beyond neoplasia (22).

Furthermore, HHV-8/KSHV develops immune evasion strategies that allows its persistence, the establishment of latency and driving oncogenesis, in the context of compromised immune surveillance (23–25). HHV-8/KSHV ensures its survival and proliferation in the host by promoting immune evasion, downregulating human leukocyte antigen (HLA) class I presentation or directly inhibiting interferon production. Specifically, HHV-8/KSHV encodes several proteins that interfere with antigen presentation. During both the latent and lytic phases, HHV-8/KSHV reduces the presence of HLA class I molecules on infected cells, impairing recognition by cytotoxic T lymphocytes (CTLs) and thus reducing immune clearance. In addition, HHV-8/KSHV encodes viral interferon regulatory factors (vIRFs) that directly inhibit type I interferon signaling. This results in the suppression of interferon-stimulated gene expression and attenuates the host’s innate antiviral response (23–25).

Therapeutically, these insights rationalize strategies that reduce inflammatory signaling and viral lytic drive alongside cytotoxic or antiangiogenic approaches: immune reconstitution or immunosuppression reduction in iatrogenic settings, targeting IL−6/IL−1 pathways, COX−2/arachidonate metabolism, or kinases like MAP4K4, and antivirals in selected contexts, in addition to standard KS treatments. The inflammation–latency–lytic triad is thus central to KS biology and to complications observed after transplantation and HHV-8/KSHV primary infection.

### 2.2 Prevalence of HHV-8 infection

The HHV-8/KSHV seroprevalence varies across different geographic regions and risk groups. Seroprevalence ranges from 3 to 7% in the United States, 5% in North Europe, 5–20% in the Mediterranean, and >50% in sub-Saharan Africa. However, even in areas where HHV-8 infection is relatively uncommon, certain groups may show high rates of seroprevalence; for example, in the United States, it ranges between 38% and 80% in people with HIV and men who have sex with men (MSM).

Currently, available guidelines do not recommend serological screening for HHV-8/KSHV before transplantation, and, consequently, data on seroprevalence are sparse in the SOT population (**Table 1**). A study on SOT patients in northern Italy (32) found a seroprevalence rate of 4% in donors and 18% in recipients. A similar discrepancy between donors and recipients was found in our study performed in southern Italy (12): HHV-8/KSHV antibodies were detected in 3.3% of donors and 8.4% recipients, with higher rates observed in liver recipients (10%) as compared to other organs (lung 6.9%, heart 6.6%, kidney 6.3%).

**Table 1:** HHV-8/KSHV seroprevalence in solid organ transplant recipients.

Author, Journal, Year, Ref	Country or Continent	Seroprevalence (%)	Type of transplant	Type of test used
Jenkins FJ. et al., *J Infect Dis*, 2002 (26)	US	Overall 20%, liver 21.7%, kidney 15.8%, heart 23%, lung 20%, multi-organ 16.7%	Multi-organ	IFA Lytic
Marcelin AG. et al., *Liver Transplant*, 2004 (27)	France	Donors 3.3%, Recipients 2.5%	Liver	IFA
García-Astudillo LA. et al, *Transpl Immunol*, 2006 (28)	Spain	Kidney 0.6%, Liver 3.4%, Northern Spain (6.6-6.9%), Central Spain (2.9%), Southeastern Spain (1.4%)	Liver and Kidney	IFA, ELISA
Frances C. et al., *Am J Transplant*, 2009 (29)	France	Donors 1.1%, Recipients 3.2%	Kidney	IFA
Pietrosi G. et al., *Am J Transplant*, 2011 (30)	Italy	Donors 4.4%, Recipients 10.2%	Liver	ELISA and IFA
Lebbe C. et al., *Am J Transplant*, 2013 (31)	Italy	Donors 12%, Recipients NA	Liver, Kidney, Heart	IFA
Chiereghin A. et al., *Transplantation*, 2017 (32)	Italy (North central)	Donors: 4%, Recipients: 18%	Kidney, Liver, Heart	IFA Lytic and ELISA
Durand C. et al., *Am J Transplant*, 2022 (33)	US	HIV+ Liver Recipients: 21% HIV+ Donors: 25% HIV false positive Donors: 0%	Liver	IFA
Mularoni A., *Am J Transplant*, 2025 (12)	Italy	Donors 3.3 %, Recipients 8.4%	Kidney, Liver, Heart, Lung	IFA
Nambiar P., *Clin Infect Dis*, 2025 (34)	US	HIV+ kidney Recipients: 40.6 % HIV+ Donors: 25.2 % HIV– Donors: 7.7 %	Kidney	Bead-based multiplex Assay
Bonazzetti C. et al., *Clin Microbiol Infect*,2025 *(*35)	Systematic review	Estimated seroprevalence: Donors 3.6%, Recipients 5.8%	Multi-organ	Mixed

ELISA, enzyme-linked immunosorbent assay; IFA, Immunofluorescence assay.

In a study conducted in four transplant centers in Spain in 2006 (28), seroprevalence was higher in liver (3.4%) than in kidney recipients (0.6%) and, among liver recipients, it was higher in the northern (6.6-6.9%) than in the central (2.9%) or the southeastern (1.4%) areas of Spain.

In liver transplant recipients, a study performed in France by Marcelin et al. (27), described a seroprevalence rate of 3.3% among donors and 2.4% among recipients while a study from Italy by Pietrosi et al. (30), reported rates of 4.4% and 10.2% among donors and recipients, respectively. In a French cohort of kidney transplants (29), the prevalence of HHV-8/KSHV antibodies was 1.1% in donors and 3.2% in recipients.

Lebbe et al. (31), in a cohort study conducted from 2004 to 2005, reported that the seroprevalence among donors was 12% (using both indirect lytic and latent immunofluorescence assay), with rates of transmission to recipients between 23% and 33%, with no differences among organ types.

Jenkins et al. (26), in a longitudinal study of HHV-8/KSHV seroprevalence conducted among 100 pediatric and adult SOT recipients in North America, documented a significant increase between pre- (5.3%) and post-transplantation (15.8%).

Bonazzetti et al. (35), in a recent systematic review, evaluated 91 studies encompassing 337 cases of KAD in SOT recipients. Serological data were available in 24% of these studies. In those studies, the authors estimated an overall median seroprevalence of 3.6% and 5.8% among donors and recipients, respectively. Rates of KAD were 18.2% among D+/R- and 7.6% among R +.

Nambiar et al. (34) recently described within the HOPE cohort a higher seroprevalence among HIV-positive kidney recipients (40.6%) than in HIV-positive donors (25.2%). MSM status was associated with higher risk of HHV-8/KSHV seropositivity. Notably, among donors without HIV the authors found a HHV-8/KSHV seroprevalence of 7.7%, like historic blood donor cohorts. In another study of the HOPE cohort (33), the authors reported a seroprevalence of 21% among HIV-positive liver transplant recipients.

An international survey (36) conducted in 2019 across 51 transplant centers in 15 countries revealed that HHV-8/KSHV serological screening is performed before or immediately after transplant in 33% of the centers, while only 8% of centers screen both donors and recipients. HHV-8/KSHV serological results were not used to determine transplant eligibility in any center. Notably, most centers (67%) reported to have diagnosed HHV-8/KSHV-related diseases after SOT.

## 3 Kaposi sarcoma

KS is the most common HHV-8/KSHV-associated neoplasm and it may involve skin, mucosa, lymph nodes, and visceral organs.

Five epidemiologic forms of KS are recognized. Classic or sporadic KS observed mainly in elderly men in the Mediterranean area and characterized by cutaneous manifestations and good prognosis. Endemic KS that is common in sub-Saharan Africa and can have a more aggressive course with lymph nodes and visceral organs involvement. Epidemic KS occurs in individuals with HIV often with disseminated disease. Post-transplant (iatrogenic) KS affects patients with iatrogenic immunosuppression such as SOT recipients and is the result of antirejection treatment and HHV-8/KSHV infection (37). Recently, in a retrospective cohort study at Memorial Sloan Kettering Cancer Center, Morales et al. (38) identified and described a fifth epidemiologic subtype of KS occurring in HIV-negative MSM without evident causes of immunodeficiency. This clinical form generally follows a slow and indolent course, and initially conservative management is usually sufficient.

Nevertheless, all KS forms, although histologically indistinguishable, differ in epidemiology, clinical characteristics, and prognosis. Immunosuppression is crucial for the development of the majority of KS forms, including the classic and endemic types, which arise in the context of “immunosenescence” and malnutrition, respectively (14).

In the SOT setting, the type of transplanted organs, the degree and duration of immunosuppression and the donor and recipient HHV-8/KSHV serostatus influence the incidence and clinical course of PT-KS.

### 3.1 Epidemiology and risk factors for KS

Globally, the distribution of KS mirrors the prevalence of HHV-8/KSHV and is more common in men than in women. In the SOT setting, the risk of KS is nearly 400 times greater than in the general population (37, 39, 40). While the incidence of epidemic KS has markedly decreased since the introduction of highly active antiretroviral therapy (HAART) (41–43), the incidence of PT-KS has shown a relative increase, due to the growing population of SOT recipients with prolonged life expectancy. Nonetheless, PT-KS remains less common than HIV-associated KS. According to a recent meta-analysis conducted by Liu et al. (44), the absolute incidence of KS is 480 per 100,000 person-years in HIV-positive patients, 68 per 100,000 person-years in SOT recipients, and 1.5 per 100,000 person-years in the general population. In US, Mbulaiteye et al. (45) reported an incidence of approximately 8.8 per 100,000 person-years among SOT, while a multicenter study conducted in Italy by Tessari et al. (46) found a higher incidence rate (2.3 cases per 1,000 individuals per year), possibly reflecting higher HHV-8/KSHV prevalence in Southern Europe.

Regarding organ types, some authors reported higher incidence of KS among lung transplant recipients (45, 47, 48). An Italian multicenter cohort (49), including more than 4,000 SOT patients, reported 73 cases of KS; factors associated with KS were male sex, older age, southern Italian origin, and lung transplantation, while a reduction of risk was observed 18 months PT, when the intensity of immunosuppression is decreased.

Regarding pre-transplant HHV-8/KSHV serostatus, some authors reported an increased risk of KS in patients with donor-derived infection but most of these data come from case series.

In the recent review, Bonazzetti et al. (35), analyzed the studies reporting both KS and serological data and described in the D+/R- group, 18 cases of KS (17 visceral KS and 1 case of only cutaneous KS), occurring after a median of 7 months after transplant, while among positive recipients (R+), 5 cases of visceral KS, occurring 12 months after transplantation.

In the study conducted by our group (12), the three cases of KS identified in HHV-8/KSHV seropositive recipients occurred at a median of 383 days after transplant. Conversely, among seronegative recipients (D+/R- or D-/R-), the two cases of concomitant KS and KICS were diagnosed earlier, at 132 and 232 days after transplantation, respectively.

Recently Ahmad et al. (50) conducted a single-center retrospective cohort study to explore the occurrence of KS in lung transplant recipients receiving organs from Public Health Service (PHS) increased-risk donors, with particular attention to the potential role of donor-derived HHV-8 infection. Between 2019 and April 2024, 90 lung transplants from increased risk donors were per performed at the study center. The most common criteria for increased risk were incarceration and injection or inhalation drug use. Among these donors, 13 (14.4%) were men who have sex with men (MSM). Notably, four recipients (30.8%) who received lungs from MSM donors developed localized KS at a median of 12 months after transplantation. None of these recipients had identifiable pre-transplant risk factors for HHV-8 raising concern for donor-derived infection, even though pre-transplant HHV-8 serology was not available for the donors or recipients.

### 3.2 Clinical characteristics, diagnosis and outcomes

**Figure 1:**
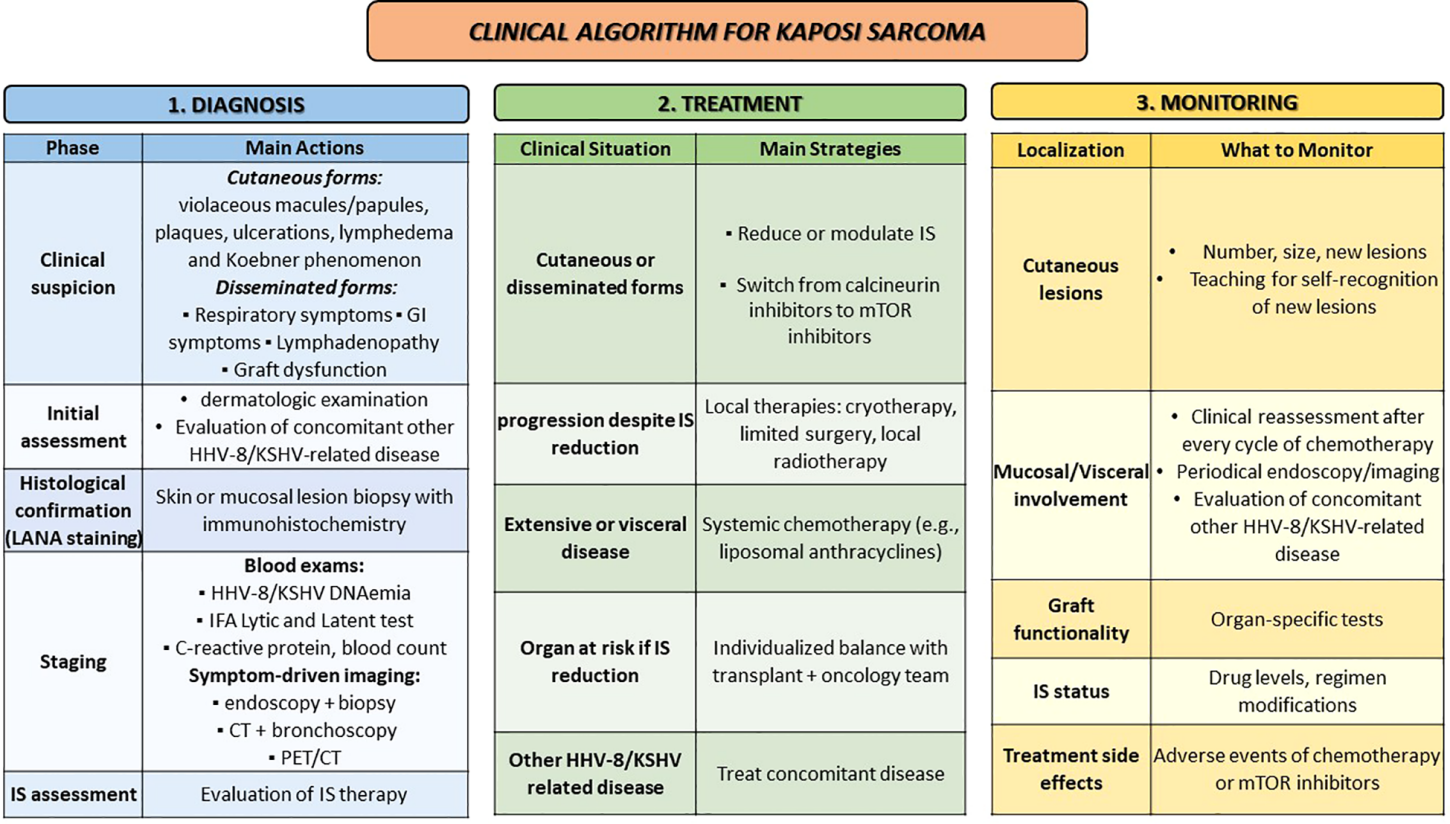
Clinical algorithm for Kaposi sarcoma. GI, gastrointestinal; IFA, immunofluorescence assay; IS, immunosuppression; mTOR, mammalian target of rapamycin.

The most common clinical manifestations of KS are painless, single or multiple violaceous or purplish non-blanching lesions (macules, papules or nodules), raised or flat that appear in the skin mainly of the lower limbs with associated lymphedema (14, 15, 51). Any part of the skin can be involved, including surgical scars, the so called “Koebner phenomenon”. KS can also affect the mucosa of the hard palate and genitals. Sometimes lesions may merge to form large plaques or form ulcerated, bleeding and painful nodules (**Figure 1**).

The diagnosis of cutaneous KS is usually based on clinical examination but, in some circumstances, it can be challenging. For instance, Kates et al. (15) present a case of KS in a patient of color, underscoring the diagnostic challenges that arise from the underrepresentation of skin of color dermatologic reference images collections. Reduced clinical suspicion in the HAART era, when numbers of KS cases in patients with HIV have declined, represents another potential pitfall.

Therefore, for the definitive diagnosis histology is warranted. At histopathological examination, KS lesions are characterized by the presence of spindle-cells, neoangiogenesis, macrophages with hemosiderin deposits and inflammatory infiltrate composed of lymphocytes and plasma cells. Spindle- cells are positive for the latency-associated nuclear antigen (LANA) of HHV-8/KSHV, identified by immunohistochemical staining (**Figure 1**).

In PT-KS, involvement of lymph nodes is present in 20-40% of cases, visceral involvement in 20-50% of cases, usually with gastrointestinal, respiratory tract or graft involvement (37). Visceral KS without skin lesions is not uncommonly reported in SOT recipients.

Diagnosis of disseminated KS requires high clinical suspicion and expertise: in the case of SOT recipients with respiratory or gastrointestinal (GI) symptoms such as shortness of breath, cough, or hemoptysis, GI bleeding or weight loss, or in the case of graft dysfunction KS should be considered in the differential diagnosis. In these cases, bronchoscopy, endoscopic evaluation of GI tract and CT-scan may be useful for both diagnosis and staging. For pulmonary lesions, the risk-benefit balance of a biopsy should be assessed due to the high risk of bleeding. Instead, suspected KS lesions in the GI tract should be biopsied for histopathological confirmation (16, 51) (**Figure 1**). Positron emission tomography/computed tomography using F-18-Fluorodeoxyglucose (FDG PET/CT) may have a role in assessing the localization of KS and guide bioptic procedures (52).

The Disease Transmission Advisory Council (DTAC) of the Organ Procurement and Transplantation Network (OPTN) identified 6 cases of KS resulting from HHV-8 transmission from deceased organ donors between July 2018 and January 2020 (47). Among 22 organ recipients from the six donors retrospectively identified as HHV-8/KSHV positive, by either HHV-8 antibody or PCR, 14 recipients had evidence of PT HHV-8/KSHV infection, six developed KS at a median time of 7.8 months PT. In detail, two lung recipients developed an aggressive KS at 10 and 12 months after transplant respectively, with rapid onset of symptoms involving the graft, lymph nodes, and other organs. Both cases were characterized by poor response to reduction of immunosuppression and systemic therapy and were rapidly fatal. In other 2 lung recipients, KS of lung and lymph nodes was diagnosed earlier (at 5 and 6 months after transplant, respectively) and treated with reduction of immunosuppression and/or switch to sirolimus and systemic chemotherapy with complete resolution and good clinical conditions at 18 months follow up. One liver recipient developed disseminated KS of the lymph nodes at 11 months after transplant with rapid worsening condition and died. One kidney transplant recipient developed KS of the graft and lymph nodes at 5 months after transplant; he underwent transplant nephrectomy but died 1 month later, for metastatic disease while awaiting chemotherapy. All these patients had visceral KS in the absence of skin lesions.

Lung transplant recipients appeared to be at higher risk of KS and the mortality rate was very high (67%) probably because of profound immunosuppression and delayed recognition and treatment (47).

In the study by Mularoni et al. (12), conducted among 1,856 recipients over a period of 12 years, five patients developed KS. Two of them had a primary infection: one case of disseminated KS with concurrent KICS occurred in a lung transplant recipient 5 months after transplant because of a donor-derived infection (D+/R-) with fatal outcome. Another case of cutaneous and gastrointestinal KS was diagnosed 11 months after liver transplant in a patient with non-donor derived primary infection (D-/R-); he was treated with switch to mammalian target of rapamycin (mTOR) inhibitors and liposomal doxorubicin B and is alive and well, at 2 years of follow up. In the cohort of seropositive recipients (R+), three cases of KS were described and occurred at a median of 383 days after transplant: among 10 seropositive heart recipients, 2 (20%) developed lymph node and visceral KS with fatal outcome, while one lung recipient with cutaneous KS was successfully treated with conversion to mTOR inhibitors.

Ocweja et al. (53) described a case of a HHV-8/KSHV donor-derived infection in 11 month-old baby that developed KS with liver nodules and portal vein thrombosis at 3 months after left lateral segment liver transplant. She was treated with sirolimus and paclitaxel with complete resolution. The recipient of the right segment developed, four months after transplant, multiple nodules in the liver allograft that were biopsied and revealed KS because of a donor-derived infection. He was treated with immunosuppression withdrawal but developed acute cellular rejection and then sirolimus was introduced but treatment with mTOR inhibitors did not lead to complete resolution. Following 13 cycles of liposomal doxorubicin without adverse effects a complete resolution of liver KS was obtained.

A case of tonsillar KS was described in a kidney transplant recipient with onset 16 months after transplant. He was treated with bilateral tonsillectomy and immunosuppression reduction and sirolimus, with KS resolution (54). Nair and colleagues (55) described a case of kidney transplant recipient with urinary symptoms and bladder wall thickening, and pelvic lymphadenopathy, ultimately diagnosed as visceral KS, without skin involvement, at 7 months after transplant. The patient was treated with pegylated lyposomal doxorubicin with complete resolution. In another renal transplant recipient (56), KS manifested as an infiltrative kidney lesion with lymphadenopathy without any skin signs, occurring about 10 weeks after transplant, necessitating graftectomy.

Cases of KS associated with Hemophagocytic lymphohistiocytosis (HLH) have been described among pediatric liver transplant recipients; in these case reports, despite aggressive course, all patients survived (53, 57, 58). HHV-8/KSHV-related HLH has also been described in adult SOT recipients, either associated with KS (59) or as the sole clinical manifestation of newly acquired HHV-8/KSHV (60), or following KICS (11, 61), with variable outcome.

Durand et al. (33), in the multicenter study conducted from 2016 to 2019, including 45 liver transplant recipients with HIV, described three cases of KS (one cutaneous KS, one KS with liver and lung involvement, and one HHV-8/KSHV-related large B cell lymphoma with KS in lymph nodes; the latter with fatal outcome) among recipients from HHV-8/KSHV seropositive donors.

In the recently published multicentric study of 418 HIV-positive kidney transplant recipients, Nambiar and colleagues (34) described 4 cases of KS: three cutaneous KS (two of them resulting from viral reactivations and one with unknown serostatus but with described risk factors for KS), one KS of the graft (donor-derived). These cases occurred at a median of 18 months after transplant and all patients recovered. The results of these studies suggest a different risk of developing KS in the different types of transplanted organs.

In a recent case series from a single center in Italy (35), over a 8-year period, a total of 12 SOT recipients were diagnosed with HHV-8/KSHV related disease, 7 of whom developed KS. The KS onset ranged from 5 to 19 months from transplant, most of them had visceral or lymph nodal involvement, and only 2 patients had cutaneous lesions. Serology was not available for all donors, and it was performed only in 4 recipients, at pre-transplant, and resulted negative. Two of these patients with KS had concurrent KICS.

In the study from Ahmad and colleagues (50), four lung transplant recipients developed localized KS with no evidence of extrapulmonary disease. In three patients lesions were detected incidentally on surveillance imaging and management relied on reduction of immunosuppression and switch to mTOR inhibitors. One patient developed severe dyspnea and a systemic syndrome with hypotension and neurological symptoms that can be possibly classified as KICS. This patient received 8 cycles of liposomal doxorubicin. All patients survived. The authors also performed a literature review, identifying 23 additional cases of PT-KS, and a pooled survival analysis that showed a significantly poorer survival in patients with disseminated KS compared with localized disease (182 vs. 563 days; p = 0.011). These cases also highlight the importance of early diagnostic vigilance; in fact, as described in this study, incidental pulmonary nodules even in asymptomatic patients should be promptly investigated with bronchoscopy and biopsy to rule out KS especially in case of PHS increased-risk donors.

In summary, PT-KS, as compared to other KS forms, may be characterized by atypical localizations, visceral involvement, in absence of cutaneous lesions and a more aggressive course, depending on the intensity of immunosuppression and serological status of donor and recipient: in seronegative recipients the lack of previous immunity could predispose the immunosuppressed host to develop a primary infection, with a more severe and rapidly disseminated disease.

SOT recipients with severe KS may develop concurrent KICS or other KADs. Therefore, worsening in disease burden or symptoms during KS presentation should alert clinicians to urgently assess for other KADs or potential causes of organ failure, such as HLH, to guide proper treatment.

While there is staging classification for epidemic (AIDS associated) KS developed by AIDS Clinical Trials Group Oncology Committee (62), currently, a similar staging system for PT-KS is lacking.

Bettuzzi et al. suggest that patient management should be tailored by considering three different clinical scenarios: localized non-aggressive, locally aggressive and disseminated KS. For PT-KS evaluation and staging, workup should be discussed on an individual basis, depending on the symptoms, degree of immunosuppression, timing from transplant and the extension of the disease. The staging workup proposed by Bettuzzi et al. (51) suggests, in the case of histologically confirmed KS, to proceed to screen the patient with all skin and mucosae inspection, node palpation and investigate melena, hematemesis, dyspnea and hemoptysis. These symptoms should guide visceral involvement assessment with CT-scan and endoscopy.

Blood exams, although generally not useful for the diagnosis, may reveal visceral involvement or graft dysfunction. HHV-8/KSHV DNA detection in lesions by polymerase chain reaction (PCR) has high sensitivity and specificity. HHV-8/KSHV detectable DNAemia has been correlated with the progression of KS but the sensitivity and specificity of DNAemia in clinical practice appear to be lower and PCR is not routinely used. In our cohort, patients with KS had a lower DNAemia as compared with those with KICS (12).

High HHV8/KSHV DNAemia and C-reactive protein levels, with or without cytopenia, could suggest that another KAD, like MCD or KICS, could be concurrently present. PEL should be suspected in patients with KS who have unexplained ascites, pericardial or pleural effusions (51).

### 3.3 Treatment and prevention

The treatment of PT-KS depends on the extension of the disease but mainly relies on modulation/reduction of immunosuppression, which is considered the essential approach (**Figure 1**). This strategy often results in the regression of KS cutaneous lesions, through reestablished control of HHV8/KSHV infection. Alongside immunosuppression tapering, conversion from calcineurin inhibitors (CNIs) to mTOR inhibitors, such as sirolimus or everolimus, is advisable, due to the direct anti-proliferative and anti-angiogenic effect and recovery of specific HHV8/KSHV T cell responses (16, 23, 37, 63–65).

Barozzi et al. (23) investigated the dynamics of HHV8/KSHV-specific T-cell responses, measured by Elispot assay, in nine SOT recipients with PT-KS. At disease onset, all patients lacked detectable T-cell responses to both latent and lytic HHV8/KSHV antigens, indicating profound impairment of virus-specific cellular immunity under CNIs-based immunosuppression. Following reduction of CNIs, restoration of HHV8/KSHV-specific T-cell was demonstrated in six renal transplant recipients, with clinical remission of KS and in two HHV-8/KSHV-seropositive renal recipients without KS. Regarding conversion to mTOR inhibitors, in two liver transplant recipients, introduction of sirolimus led to normalization of the T-cell repertoire and recovery of both effector and memory HHV-8/KSHV-specific T cells, which correlated with complete KS remission; in one renal recipient, sirolimus conversion resulted in early recovery of effector but not memory T cells and only partial KS remission was observed. These findings demonstrate that restoration of HHV-8/KSHV-specific T-cell immunity is important for KS remission in the PT setting, and that this can be achieved with careful immunosuppression modulation, including mTOR inhibitors therapy, without compromising graft survival.

For limited cutaneous disease, local therapies such as surgical excision, cryotherapy, radiotherapy, intralesional chemotherapy or local imiquimod may be considered. In more extensive or aggressively progressive KS, chemotherapy is employed with Pegylated liposomal doxorubicin or, as second line therapy, paclitaxel, with the choice guided by disease burden and patient comorbidities (51).

Most of randomized controlled trials (RCTs) were conducted in patients with HIV while in PT-KS RCTs are lacking. In a retrospective multicentric study by Delyon et al. (66), conducted between 1985 and 2011 on 145 SOT recipients with KS, the majority of patients were treated with tapering of immunosuppression, while conversion to mTOR was performed in 28% of patients and chemotherapy in only 16% (mainly with visceral disease). With this approach, complete response was achieved in 40% of SOT recipients. A study conducted in 15 kidney transplant recipients with proven KS, showed that therapy with sirolimus slows the progression of KS and prevents episodes of rejection (63).

The early timing of treatment initiation, either through adjustment of immunosuppression in localized forms or chemotherapy in the case of disseminated disease, is crucial for achieving a favorable outcome. When localized disease is promptly treated, complete response can be achieved in up to 40% of cases, whereas visceral dissemination may result in mortality rates as high as 17% (66–69). In our cohort (12) two heart recipients with visceral KS treated with chemotherapy did not survive while the lung recipient with cutaneous KS treated with conversion to mTOR inhibitors survived. Bonazzetti et al. (35), reported 147 cutaneous and 99 visceral KS. Treatment consisted of chemotherapy in 18.8% of cases, mainly doxorubicin and paclitaxel. The 90-day mortality was 19.7%, reaching 29% when non-cutaneous KS form was excluded.

In order to reduce short and long-term side effects associated with chemotherapy, some alternative strategies have been investigated such as immunotherapy (70–72). Two clinical trials evaluated the efficacy of the PD-1 inhibitor pembrolizumab for the treatment of KS. In the phase I trial conducted by Uldrick et al. (71) in a cohort of HIV patients with KS, pembrolizumab showed a manageable safety profile and some clinical benefit with partial responses and disease stabilization. The study’s findings supported further investigations. In the phase 2 multicenter study by Delyon et al. (73), pembrolizumab was used in 17 patients with classic or endemic KS and resulted in overall response rate of 71%.

A recent multicenter phase I trial of pembrolizumab in HIV-positive KS patients reported a 62% response rate, with durable responses (72).

However, checkpoint inhibitors are not yet approved for KS and carry a risk of allograft rejection in SOT recipients (51, 74, 75). Therefore, even if they present a promising investigational approach further studies are needed to define their role and safety.

Other emerging therapies for KS include immunomodulatory and antiangiogenic agents, which show promise in restoring anti-HHV-8/KSHV immunity and controlling tumor growth, though their use in PT-KS remains still investigational. Pomalidomide, an oral immunomodulatory drug that enhance T and NK cell activity and has antiangiogenic properties, has demonstrated efficacy in both HIV-associated and classic KS, and is FDA-approved for KS in patients with or without HIV (76). In a phase 1/2 study, pomalidomide achieved response rates of approximately 67% in HIV-positive patients, including those previously treated with chemotherapy. Lenalidomide, another immunomodulatory agent, has shown clinical activity in HIV-associated KS, expanding options for patients with refractory disease (77).

Cytokine-driven strategies, such as subcutaneous IL-12, aim to control disease, by stimulating immune response through increased T cell and CD4+ activity (78, 79). Unfortunately, studies did not include SOT recipients and therefore, the benefit of these novel treatments in this population has yet to be proven.

In some cases, KICS may concurrently occur with KS in SOT recipients. Recent data suggest that rituximab-based therapy, with or without anti-IL-6/IL-6R monoclonal antibodies, can be successful in patients with KICS (12). Importantly, rituximab has been associated with flares–ups of KS in HIV positive patients. Great care must be taken in the case of concurrent KICS and KS, and, if these entities coexist, rituximab and liposomal doxorubicin should be used together, following protocols used for managing concurrent KS and MCD (6, 80, 81).

Lastly, the development of an effective HHV-8/KSHV vaccine could play a pivotal role in reducing KS in population at risk and in endemic countries with limited access to cure. However, currently no vaccine is currently available for HHV-8/KSHV in clinical practice (51). The complexity of the viral lifecycle and its capacity to establish latency poses significant challenges to vaccine (82).

Similar to EBV and EBV-related PLTDs, the use of HHV-8/KSHV-specific cytotoxic T-lymphocytes could have the potential to reduce the burden of KS, by restoring and enhancing specific T cell activity (16). However, there are no T cell therapies currently available for prevention or treatment of KADs. Nonetheless, current research efforts include preclinical mRNA vaccine design identification of optimal HHV-8/KSHV antigens able to induce expansion of HHV-8/KSHV specific T cell (83, 84). HHV-8/KSHV and EBV co-infection of mice with reconstituted human immune systems (humanized mice) has been reported to lead to IgM responses against both latent and lytic HHV-8/KSHV antigens, and expansion of central and effector memory CD4+ and CD8+ T cells. Among these, HHV-8/KSHV and EBV dual infection allows for the priming of CD8+ T cells that are specific for the lytic KSHV antigen K6 and able to kill HHV-8/KSHV and EBV infected B cells, suggesting that K6 may be exploited as a vaccine antigen (85).

In conclusion, the management of PT-KS remains a clinical challenge, largely guided by case reports and expert opinions due to the lack of standardized protocols. We believe that management should be personalized, based on type of transplanted organ, degree of immunosuppression, extension and localization of KS disease and the possible occurrence of concurrent KAD should always be considered when evaluating a patient with PT-KS.

### 3.4 Kaposi sarcoma characteristics by organ type

Our literature search identified a total of 100 studies (**Table 2**). Overall, 663 cases of PT-KS were reported with most of cases described among kidney recipients (452 cases) as compared to other organs (103, 63, and 45 among liver, heart, and lung recipients). The distribution of PT-KS varies considerably across different types of transplanted organs, with distinct patterns emerging in terms of incidence and clinical manifestations. Among studies that reported clinical outcome, overall survival was 66.5% (241/362). Outcomes differed substantially across organ types: kidney and liver recipients showed the highest survival rate [70% (196/279) and 63% (27/43)]; survival decreased markedly among heart (46%, 6/13) and lung (44%, 12/27) transplant recipients (**Table 2**).

**Table 2:** KS in solid organ transplant recipients.

Author, Journal, Year, Ref	Country	Number of cases	Time from Tx	Donor-derived or reactivation	Cutaneous/Disseminated	Other KADs	Treatment	Outcome
Kidney								
Bencini PL. et al., Arch Dermatol, 1993 (86)	Italy	11	Median 29,9 months	NR	Cutaneous (4/11 cases) Disseminated (7/11 cases)	no	IS reduction or discontinuation	Complete resolution (9/11 cases)
Montagnino G. et al., Am. J. Nephrol, 1994 (87)	Italy	13	Median 38,7 months	NR	Cutaneous (5/13 cases) Disseminated (8/13 cases)	no	IS reduction or discontinuation	Complete resolution (11/13 cases)
Margolius L. et al., S Afr Med J, 1994 (88)	Johannesburg	5	NR	NR	Cutaneous (1/5 cases) Disseminated (4/5 cases)	no	IS reduction	Complete resolution (4/5 cases)
Lesnoni LP. et al, Dermatology. 1997 (89)	Italy	10	Median 23,1 months	NR	Cutaneous (5/10 cases) Disseminated (5/10 cases)	no	IS reduction, radio/CHT	Complete resolution (9/10 cases)
Bottalico D. et al., Nephrol Dial Transplant 1997 (90)	Italy	2	20 months (case 1-2)	NR	Cutaneous	no	IS reduction, antiviral	Complete resolution
Parravicini C. et al., Blood, 1997 (91)	Italy	10	Median 8,5 months	NR	NR	MCD (1 case)	IS	NR
Noel JC. et al., Transpl Int, 1997 (92)	Belgium	1	5 months	NR	Cutaneous, visceral	no	IS reduction and discontinuation, nephrectomy	Complete resolution
Qunibi Q. et al., Transplantation, 1998 (93)	Saudi Arabia	14	Median 13 months	NR	NR	NR	IS	NR
Lee PC. et al., Lancet,1998 (94)	Taiwan	1	5 months	NR	NR	no	IS	NR
Matsushima AY. et al., Am J Surg Pathol, 1999 (95)	US	1	24 months	NR	Disseminated	MCD	No specific therapy	Deceased
Gòmez-Romàn JJ. et al, Arch Pathol Lab Med, 1999 (96)	Spain	1	6 months	NR	Cutaneous	no	IS discontinuation	Complete resolution
Eberhard OK. et al., Transplantation, 1999 (97)	Germany	5	Median 48,8 months	NR	Cutaneous (3/5 cases), Disseminated (2/5 cases)	no	IS reduction, tacrolimus	Complete resolution (4/5 cases)
Nagy S. et al., Transplantation, 2000 (98)	Hungary	1	3 months	NR	Disseminated	no	IS reduction, radiotherapy, graftectomy	Complete resolution
Barete S. et al., Arch Dermatol, 2000 (99)	France	18	Median 21 months	Reactivation	Cutaneous, disseminated	no	IS reduction, cryotherapy	Complete resolution (12/18 cases)
Kapelushnik J. et al., Br J Haematol, 2001 (100)	France	1	9 months	NR	Cutaneous	no	IS discontinuation, combination CHT	Complete resolution
Andreoni M. et al., J Infect, 2001 (101)	Italy	4	NR	NR	NR	NR	NR	NR
El-Agroudy AE. et al., Am J Transplant, 2003 (102)	Egypt	24	Median 33,9 months	NR	Cutaneous (20/24 cases) Disseminated (3/24 cases), only visceral (1/24 cases)	no	IS reduction, radio/CHT	Complete resolution (12/24 cases)
Mitxelena J. et al., Int J Dermatol, 2003 (103)	Spain	6	Median 10,5 months	NR	Cutaneous (5/6 cases), Visceral (1/6 cases)	no	IS reduction, criotherapy	Complete or partial resolution (4/6 cases)
Shahbazian H. et al., Urol J, 2004 (104)	Iran	14	Median 18 months	NR	Cutaneous (11/14 cases), Visceral (3/14 cases)	no	IS reduction or discontinuation	Complete or partial remission (10/14 cases)
Boeckle E. et al., Transplant Proc, 2005 (105)	Austria	3	Median 15,7 months	NR	Cutaneous (1/3 cases) Disseminated(2/3 cases)	no	IS reduction, Switch to sirolimus, radiation	Complete resolution (2/3 cases)
Moosa MR. et al., QJM, 2005 (106)	South Africa	21	Median 32 months	NR	Cutaneous (13/21 cases), Visceral (4/21 cases), Disseminated (4/21 cases)	no	IS reduction, CHT	Complete or partial remission (16/21 cases)
Stallone G. et al., *N Engl J Med*, 2005 (62),	Italy	1	3 months	Reactivation	Cutaneous	no	Discontinuation of cyclosporine + Sirolimus	Complete resolution
Barozzi P. et al., Transplantation,2006 (107)	Italy	1	NR	Primary infection	Disseminated	no	NR	NR
Gonzalez-Lopez MA. et al., Dermatology, 2006 (108)	Spain	1	12 months	NR	Cutaneous	no	IS reduction and discontinuation of prednisone	Complete resolution
Garcia-Astudillo LA. et al., Transpl Immunol, 2006 (28)	Spain	4	Median 33,7 months	NR	Cutaneous	NR	NR	NR
Silvia S. et al., Prot J Nephrol Hypert, 2007 (109)	Portugal	6	Median 23 months	NR	Cutaneous (3/6 cases) Disseminated (3/6 cases)	no	IS reduction, switch to sirolimus	Complete resolution (5/6 cases)
Boulanger E et al., Am J Transplant, 2008 (110)	France	1	5 months	NR	Disseminated	PEL	CHT	Deceased
Dudderidge TJ. et al., Transpl Infect Dis, 2008 (111)	UK	1	12 months	Donor-Derived	Visceral, graft	no	IS reduction	Complete resolution
Einollahi B. et al., Int Urol Nephrol, 2009 (112)	Iran	55	Median 27 months	NR	Cutaneous (48/55 cases) Visceral (7/55 cases)	PTLD (1/55 cases)	IS reduction, switch to sirolimus, CHT	Complete resolution (45/55 cases)
Piselli P., et al., Transplant Proc, 2009 (49)	Italy	31	NR	NR	NR	NR	NR	NR
Yaich S., et al., Transplantation Proceedings, 2012 (113)	Tunisia	10	Median 24,7 months	NR	Cutaneous (9/10 cases) Visceral (2/10 cases)	NR	IS reduction, switch to sirolimus, CHT	Complete resolution (7/10 cases)
Prinz Vavricka BM. et al., Clin Exp Dermatolo, 2012 (114)	Switzerland	2	240 months (case1) 192 months (case2)	NR	Cutaneous (case 1), Disseminated (case 2)	no	IS reduction (case 1-2) surgical excision, radiotherapy (case1)	Complete resolution (1/2 cases)
Lebbe C., et al., Am J Transplant,2013 (31)	France	1	6 months	NR	Cutaneous	no	IS reduction, switch to sirolimus	Complete resolution
Raeisi D., et al., Int J Hematol Oncol Stem Cell Res, 2013 (115)	Iran	17	Median 18,7 months	NR	Cutaneous (12/17 cases) Disseminated (5/17 cases)	no	IS reduction, switch to sirolimus, CHT	NR
Zavos G., et al., Transplant Proc, 2014 (116)	Greece	24	Median 31,6 months	NR	Cutaneous (14/24 cases), Visceral (2/24), Disseminated (8/24 cases)	no	IS reduction, switch to everolimus, radio/CHT	Complete resolution (12/24 cases)
Cota C., et al., Am J Dermatophatol, 2014 (117)	Austria	1	60 months	NR	Cutaneous	no	IS, radiotherapy	Complete resolution
Borges-Costa J. et al., et al., BMJ, 2016 (118)	Portugal	1	12 months	NR	Cutaneous	no	IS discontinuation, doxorubicin	Complete resolution
Vijgen SMD., et al., Transplantation,2016 (119)	Switzerland	1	NR	Donor-Derived	Disseminated	MCD	IS reduction, rituximab	Deceased
Gorsane I. et al., Clin Transplant. 2016 (120)	Tunisia	12	Median 23,3 months	NR	Cutaneous (8/12 cases) Disseminated (4/12 cases)	PTLD (1/12 cases)	IS reduction, switch to sirolimus	Complete resolution (8/12 cases)
Park YJ., et al., Korean J Intern Med, 2017 (121)	Korea	1	5 months	NR	Cutaneous, skin	HLH	IS reduction, Switch to Sirolimus	Deceased
Bohelay G., et al., Clin Transplant, 2017 (122)	France	8	Median 29,7 months	NR	Cutaneous and disseminated in 7/8 cases	no	IS reduction, switch to sirolimus	Complete resolution (7/8 cases)
Chiereghin A. et al., Transplantation, 2017 (32)	Italy	1	10 months	Reactivation	Disseminated	no	IS reduction, doxorubicin	Complete resolution
Cahoon EK., et al., Int J Cancer, 2018cahoon (48)	US	91	NR	NR	NR	NR	NR	NR
Cain O., et al., Histopathology, 2018 (123)	UK	1	24 months	NR	Visceral	PEL, HLH	IS	Deceased
Nair V. et al., *Transpl Infect Dis*, 2019 (54)	US	1	7 months	Reactivation	Visceral	no	Switch to mTOR, liposomal doxorubicin, switch to mTOR	Complete resolution
Raedemaeker J. et al., BMJ, 2019 (124)	Belgium	1	60 months	NR	Cutaneous, skin and leg	no	IS reduction, doxorubicin, switch to sirolimus	Deceased
Howard J.H., et al., *Transpl Infect Dis*, 2020 (53)	US	1	16 months	Unknown	Lymph nodes, tonsils	PTLD	IS reduction, sirolimus, Bilateral tonsillectomy	Complete resolution
Dollard S.C. et al., *Am J Transplant*, 2021 (47)	US	1	5 months	Donor-derived	Visceral, allograft	no	IS discontinuation, CHT	Deceased
Story MT. et al., Transpl Infect Dis, 2021 (167)	US	1	5 months	Donor-derived	Visceral allograft lymph nodes	no	IS discontinuation, graftectomy	Deceased
Lee JJ. et al., *Korean J transplant*, 2023 (55)	Korea	1	3 months	Donor-derived	Visceral	no	graftectomy	Complete resolution
Nambiar P. et al., *Clin Infect Dis*, 2025 (34)	US	4	Median 46 months	Donor-derived (1/4 cases) Reactivation (2/4 cases)	Cutaneous (3/4 cases) Allograft (1/4 cases)	MCD	sirolimus/Rituximab, Doxorubicin	Deceased
Bonazzetti C. et al. CMI, 2025 (35)	Italy	3	Median 46 months	NR	Cutaneous (2/3 cases), Visceral (1/3 cases)	1/3 KICS	IS reduction, Doxorubicin	Complete resolution (3/3 cases)
Srinivasan S., et al., Cureus, 2026 (125)	US	1	< 12 months	NR	Visceral, allograft	no	IS reduction	Deceased
Total number of studies: 53		Overall Kidney Tx cases: 452						Survival: 196/279 (70%)
Liver								
Matsushima AY., et al., Am J Surg Pathol, 1999 (95)	US	1	2 months	NR	Visceral, allograft	MCD	No specific therapy	Complete resolution
Pozo F., et al. Transplantation, 2000 (126)	Spain	1	9 months	Donor-Derived	Disseminated	no	IS reduction	Deceased
Barete S., et al. Arch Dermatol, 2000 (99)	France	2	Median 21 months	Reactivation	Cutaneous, disseminated	no	IS reduction, cryotherapy	Complete resolution (2/2 cases)
Andreoni M. et al., J Infect, 2001 (101)	Italy	9	NR	NR	NR	NR	NR	NR
Aseni P. et al., Liver Transpl, 2001 (127)	Italy	5	Median 14,8 months	NR	Cutaneous (1/5 cases), disseminated (4/5 cases)	NR	IS reduction, CHT	Complete resolution (1/5 cases) Deceased (4/5 cases)
Marcelin AG. et al., Liver Transplant, 2004 (27)	France	2	NR	Primary Infection	Disseminated	no	IS reduction	Deceased
Akay S. et al. Transplant Proc, 2005 (128)	Turkey	1	13 months	No	Visceral	no	IS reduction, vincristine	Complete remission
Boeckle E. et al., Transplant Proc, 2005	Austria	1	19 months	NR	Disseminated	no	IS reduction	Deceased
Verucchi G. et al., Transpl Infect Dis, 2005 (129)	Italy	1	24 months	NR	Disseminated	no	IS reduction	Complete resolution
Garcia-Astudillo LA. et al., Transpl Immunol, 2006 (28)	Spain	5	Median 10,4 months	NR	Cutaneous	NR	NR	NR
Gaitonde S. et al., Histopathology, 2007 (130)	US	1	6 months	NR	Disseminated	MCD	IS reduction	Deceased
Di Benedetto F., et al. J Cancer Res Clin Oncol, 2008 (131)	Italy	6	Median 6,2 months	NR	Cutaneous (2/6 cases) Disseminated (4/6 cases)	NR	IS, switch to sirolimus, doxorubicin	Complete resolution (3/6 cases)
Piselli P. et al., Transplant Proc, 2009 (49)	Italy	8	NR	NR	NR	NR	NR	NR
Celtik C. et al., Pediatr Transplant, 2011 (58)	Turkey	1	5 months	NR	Disseminated	NR	IS reduction, CHT	Complete resolution
Benhammane H. et al., Case Rep Oncol Med., 2012 (132)	Morocco	1	36 months	NR	Visceral	no	IS reduction, switch to sirolimus	Complete resolution
Malla I. et al, Arch Argent Pediatr, 2013 (133)	Argentina	1	4 months	NR	Visceral, lymph nodes	no	IS reduction, switch to sirolimus	Complete resolution
Lebbe C. et al., Am J Transplant,2013 (31)	France	2	Median 6,5 months	NR	Disseminated	no	IS reduction, switch to sirolimus, rituximab	Complete resolution (1/2 cases)
Garzino-Demo P. et al., J Craniofac Surg, 2017 (134)	Italy	1	6 months	NR	Disseminated	no	IS reduction, rituximab	Complete resolution
Cahoon EK. et al., Int J Cancer, 2018 (48)	US	35	NR	NR	NR	NR	NR	NR
Dollard S.C., *Am J transplant*, 2018 (47)	US	1 (Liver/ Kidney)	8 months	Donor-derived	Visceral	no	switch to sirolimus	Complete resolution
Fu W. et al., *Transpl Infect Dis*, 2018 (135)	US	1	4 months	Donor-derived	Multiple nodules	no	IS reduction, doxorubicin	Complete resolution
Ocwieja KE. et al., *Pediatr Transplant*, 2019 (52)	US	1	3 months	Donor-derived	Visceral, lymph nodes	no	switch to sirolimus, paclitaxel	Complete resolution
Copeland MMM. et al., BMJ Case Rep, 2021 (136)	UK	1	5 months	Donor-derived	Disseminated	no	IS, switch to tacrolimus	Deceased
Dollard S.C. et al., *Am J Transplant*, 2021 (47)	US	1	11 months	Donor-derived	Visceral, lymph nodes	no	IS	Deceased
Marando A., et al., Phatologica, 2022 (137)	Italy	1	4 months	NR	Disseminated	no	IS reduction	Complete resolution
Zhang SH. et al., World J Clin Cases, 2022 (138)	China	1	NA	NA	Visceral, lymph nodes	PTLD	IS reduction, rituximab	Complete resolution
Durand CM. et al., *Am J Transplant*, 2022 (33)	US	3	Median 7,6 months	Donor-derived	Cutaneous, Disseminated	B-cell Lymphoma	IS reduction, tacrolimus	NR
Cordeiro C., et al., Pediatr Transplant, 2023 (57)	Portugal	3	Median 11,7 months	NR	Visceral (1/3 cases), disseminated (2/3 cases)	MCD (1/3), HLH (2/3) PTLD (1/3)	IS reduction/discontinuation, sirolimus, CHT	Complete resolution3/3 cases)
Kates O. et al, *Transpl Infect Dis*, 2024 (16)	US	1	4 months	Donor-derived	Disseminated	no	switch to sirolimus	Deceased
Clemmenson JLR. et al., IDCases, 2025 (139)	Denmark	1	5 months	Donor-derived	Visceral, allograft	no	IS reduction, switch to everolimus	Complete resolution
Mularoni A. et al, *Am J Transplant*, 2025 (12)	Italy	1	8 months	Primary infection non DDI	Disseminated	KICS, HLH	IS reduction, switch to mTOR, CHT, Rituximab	Complete resolution
Bonazzetti C. et al. CMI, 2025 (35)	Italy	3	Median 8 months	NR	Disseminated (2/3 cases)	1/3 KICS	IS reduction, Doxorubicin	Complete resolution (3/3 cases)
Total number of studies: 32		Overall liver Tx cases: 103						Survival: 27/43 (63%)
Heart								
Jones D. et al, NEJM, 1998 (140)	US	1	5 months	NR	Visceral	PEL	IS reduction, CHT	Deceased
Mendez JC. et al., Clin Transplant, 1999 (141)	US	1	3 months	NR	Disseminated	no	IS reduction	Deceased
Barete S. et al., Arch Dermatol, 2000 (99)	France	1	Median 21 months	Reactivation	Cutaneous, disseminated	no	IS reduction, cryotherapy	Complete resolution
Emond JP. et al., J Clin Microbiol, 2002 (142)	France	1	7 months	Reactivation	Cutaneous, skin	no	NR	NR
Collart F. et al., Transplant Proc. 2004 (143)	France	1	21 months	NR	Visceral, gum and tonsillar	no	IS reduction, rituximab	Deceased
Boeckle E. et al., Transplant Proc, 2005 (105)	Austria	1	2 months	NR	Cutaneous	no	IS reduction, Switch to sirolimus, Surgery, radiation	Complete resolution
Wasywich CA. et al., J Heart Lung Transplant. 2006 (144)	New Zealand	1	1,2 months	NR	Cutaneous	no	IS modification, sirolimus. radiotherapy	Complete resolution
Piselli P. et al., Transplant Proc, 2009 (49)	Italy	27	NR	NR	NR	NR	NR	NR
Patel A. et al., Heart Lung, 2014 (145)	US	1	NR	NR	Visceral, lymph nodes	MCD	IS reduction, Switch to sirolimus, rituximab	Deceased
Cahoon EK. et al., Int J Cancer, 2018 (48)	US	22	NR	NR	NR	NR	NR	NR
Kugasia IAR. et al., Transpl Infect, 2019 (146)	US	1	5 months	NR	Cutaneous	PEL	IS reduction, Switch to sirolimus	Deceased
Antonio R. et al., Transpl Infect Dis, 2021 (147)	Italy	1	11 months	Donor-derived	Visceral, lymph nodes, GI	KICS	IS reduction, Switch to mTOR, doxorubicin	Complete resolution
Mularoni A., *Am J Transplant*, 2025 (12)	Italy	2	Median 12,5 months	Reactivation	Visceral	no	IS reduction, switch to mTOR, CHT, Rituximab	2/2 Deceased
Kumar A., et al., Eur J Heart Fail, 2025 (148)	US	1	5 months	NR	Cutaneous	no	IS reduction, switch to sirolimus	Complete resolution
Bonazzetti C. et al. CMI, 2025 (35)	Italy	1	14 months	NR	Disseminated	KICS	IS reduction, switch to mTOR, doxorubin	Complete resolution
Total number of studies: 15		Overall heart Tx cases: 63						Survival: 6/13 (46%)
Lung								
Sleiman C. et al., Eur Respir J, 1997 (149)	France	1	6 months	NR	Visceral	no	IS reduction	Complete resolution
Schsenberg-Studer EM. et al., J Am Acad Dermatol, 1999 (150)	UK	1	8 months	Reactivation	Disseminated	no	IS reduction, vryotherapy	Deceased
Huang PM. Et al., Transplant Proc, 2003 (151)	Taiwan	1	17 months	NR	Disseminated	no	IS reduction	Complete resolution
Sathy SJ. Et al. Am J Transplant, 2008 (152)	US	2	Median 6 months	NR	Cutaneous	no	IS reduction, switch to mTOR, doxorubicin, rituximab	Deceased (2/2 cases)
Martinez S. et al, J Thorac Imaging, 2008 (153)	US	2	Median 6 months	NR	Cutaneous (1/2 cases) Visceral (1/2)	no	NR	Deceased (1/2) NR (1/2)
Piselli P. et al., Transplant Proc, 2009 (49)	Italy	7	NR	NR	NR	NR	NR	NR
Santos-Antunes J. et al., J Gastrointestin Liver Dis, 2015 (154)	Portugal	1	NA	NR	Disseminated	NR	Switch to mTOR	NR
Meira L et al., Arch Broncopneumol, 2017 (155)	Portugal	2	Median 30 months	NR	Disseminated skin and pulmonary (case1), gastrointestinal (case2)	NR	None (case1), IS reduction, mTOR (case2)	Deceased (2/2 cases)
Nannini N. et al., Exp Clin Transplant, 2017 (156)	Italy	1	22 months	NR	Visceral	NR	IS suspended, doxorubicin	Deceased
Salguero F. et al., Actas Dermosifiliogr, 2017 (157)	Spain	1	7 months	Donor-derived	Cutaneous	NR	IS reduction, mTOR, doxorubicin	Complete resolution
Cahoon EK. et al., Int J Cancer, 2018 (48)	US	9	NR	NR	NR	NR	NR	NR
Ng J. et al, J Heart Lung Transplant, 2018 (156)	US	1	15 months	NR	Visceral	NR	Palliative care	Deceased
Galluzzo Mutti L. et al., Pediatr Transplant,2019 (158)	Argentina	1	8 months	NR	Disseminated	NR	IS reduction, switch to mTOR	Deceased
Dollard S.C. et al., *Am J Transplant*, 2021 (47)	US	4	Median 7,7 months	Donor-derived	Visceral (2/4 cases) disseminated (2/4 cases)	no	IS discontinuation, switch to sirolimus, doxorubicin	Complete resolution (2/4 cases) Deceased (2/4 cases)
Turkkan S. et al., Exp Clin Transplant, 2022 (159)	Turkey	1	8 months	NR	Visceral	NR	Surgery, CHT	Complete resolution
Van Gemert JP. Et al., J Heart Lung Transplant, 2022 (160)	Netherlands	1	5 months	Donor-derived	Disseminated	NR	IS reduction, switch to mTOR, doxorubicin, valganciclovir	Deceased
Trabzonlu L. et al., Diagn Cytopathol, 2024 (161)	US	1	11 months	NR	Visceral	NR	IS reduction, switch to mTOR	Complete resolution
Nathani A. et al., Transplant Proc, 2024 (162)	US	2	Median 15 months	NR	Visceral	NR	IS reduction and CHT	Deceased (2/2 cases)
Mularoni A. et al., *Am J Transplant*, 2025 (12)	Italy	2	Median 8 months	1 Donor-derived and 1 reactivation	1 Cutaneous and 1 visceral	1/2 KICS	IS reduction, switch to mTOR, CHT, Rituximab	Complete resolution (1/2 cases)
Ahmad S. et al., et al., J Heart Lung Transplant, 2026 (50)	US	4	Median 12,5 months	NR	Visceral	NR	IS reduction, Switch mTOR, (all patients) radiation (case1) and doxorubicin (case2)	Complete resolution
Total number of studies: 20		Overall lung Tx cases: 45						Survival: 12/27 (44%)

CHT, chemotherapy; IS, immunosuppression; KICS, Kaposi’s Sarcoma-associated Herpesvirus Inflammatory Cytokine Syndrome; KS, Kaposi Sarcoma; MCD, Multicentric Castleman Disease; mTOR, mammalian target of rapamycin; PTLD, Post-transplant lymphoproliferative disorder.

## 4 Gaps of knowledge and future directions

Despite advances in supportive care and the implementation of HHV-8/KSHV–targeted therapies that have improved outcomes in many cases of HHV-8/KSHV-associated diseases, mortality in SOT recipients remains high. Moreover, while the incidence of AIDS-associated KS is decreasing, the incidence of PT-KS is expected to rise in the coming years, as the population of immunocompromised hosts expands, largely due to the broader criteria for organ transplantation, increased life expectancy, and the heightened risk of neoplasms associated with aging and immunosenescence.

In our view, Moore and Chang (163) offered the most compelling definition of KS in their recent review on viral oncogenesis: “KS, a cancer, behaves like an infectious disease, which, of course, it is, as well.” Consistent with this, standardized incidence ratios for various cancers among transplant patients, compared to the general population, show that KS is extreme in its predilection for immunosuppressed patients, while other viral-related cancers result less significantly increased in immunocompromised populations (163). Despite growing knowledge, much about the reciprocal influence by HHV-8/KSHV and infected host factors remains to be clarified.

The pathogenesis and distribution of KS appear to be shaped by a multifactorial interplay of genetic, immunological, and environmental influences as witnessed, for instance, by the absence of KS in HIV-positive individuals with Hemophilia A. This could be related to the X-linked defect in factor VIII, synthesized by endothelial cells; this defect could lead to an altered endothelial environment that is less permissive to HHV-8/KSHV infection and subsequent tumor development.

Similarly, endemic KS in children often presents with predominant lymph node involvement. This pattern may reflect the immune landscape in children with malaria, who exhibit elevated levels of M2-polarized monocytes and macrophages, cells highly susceptible to HHV-8/KSHV infection, which produce B cell–activating factors (BAFF, APRIL), creating a supportive niche for viral latency and B cell proliferation, and likely contributing to both the localization and geographic distribution of KS (164). Another interesting observation is that KS rarely involves the central nervous system, possibly due to limited trafficking of infected or permissive immune cells to this compartment (165). Moreover, mechanisms of oncogenic and inflammatory processes, pathological angiogenesis, and immune evasion remain poorly characterized and represent important areas for further research. Therefore, advancing our understanding of the complex interplay between the virus and host biology is critical.

Diagnosis and clinical management of KS and other KADs in SOT recipients are further challenged by atypical disease presentations and frequent occurrence in patients who are acutely ill and prone to a rapid clinical decline. Novel diagnostic approaches are currently under evaluation, including cytokine profiles and specific T cell response patterns associated with HHV-8/KSHV-related conditions, to help predict patients’ clinical outcomes. A study by Busà et al (13)., who evaluated cytokine expression and specific T cell responses in recipients with HHV-8/KSHV infection, found that those with KS exhibited cytokine profiles indicative of immune exhaustion, whereas recipients with KICS showed patterns of immune activation.

## 5 Public health considerations for donor and recipient screening

Recipients with HHV-8/KSHV mismatch and recipients with pre-transplant positive HHV-8/KSHV serology are at high risk for HHV-8/KSHV associated complications (12, 135, 166). This underscores the urgent need to explore the usefulness of serological screening of donors and recipients, to put in place early, individualized, and effective preemptive strategies, and establish protocols for rapid diagnosis. Reliable HHV-8/KSHV serological screening prior to transplantation, alongside consideration of HHV-8/KSHV DNA monitoring, may help identify and monitor high-risk individuals.

From a public-health perspective, the expanding reliance on PHS increased-risk donors may unintentionally increase exposure to pathogens that are not routinely addressed by current screening programs, including HHV-8/KSHV. Therefore, universal screening of donors and recipients may be an effective strategy for post-transplant risk mitigation. In countries with expected low seroprevalence or where universal serological screening is not feasible, it can be considered to adopt a risk-based strategy with screening of PHS increased-risk donors, particularly at centers with high PHS increased-risk donor utilization. Given ongoing donor shortages, avoidance of PHS increased-risk donors is neither feasible nor desirable; however, greater awareness and targeted risk-based strategies may allow earlier identification and intervention.

## 6 Conclusion

KS remains a significant challenge in SOT recipients, with high mortality in this vulnerable population. Lack of clinical expertise and low index of suspicion may contribute to missed or delayed diagnosis and increased mortality. Therefore, increasing clinician awareness is essential.

Serological screening of donors and recipients, although not recommended by current guidelines due to the lack of standardized assays, could serve as a valuable and innovative tool for risk stratification and could allow early diagnosis in the clinician aware of D+/R- or R+ with symptoms suggestive of KS.

To evaluate the risk/benefit balance related to serological screening, multicenter studies involving worldwide-distributed transplant centers are needed, with the primary objective of developing a proactive risk mitigation approach, based on serological screening of both donors and recipients, along with targeted clinical and virological monitoring of high-risk patients.

## Glossary

CTLs: cytotoxic T lymphocytes
CNI: Calcineurin inhibitor
D: donor
FDG PET/CT: Positron emission tomography/computed tomography using F-18-Fluorodeoxyglucose
GI: gastrointestinal
GLPD: germinotropic lymphoproliferative disorder
HHV-8/KSHV: Human herpes virus 8/Kaposi’s sarcoma-associated herpesvirus (HHV-8/KSHV)
HAART: Highly active antiretroviral therapy
HLA: human leukocyte antigen
LANA: latency-associated nuclear antigen
KAD: HHV-8/KSHV-associated disease
KICS: Kaposi’s Sarcoma-associated Herpesvirus Inflammatory Cytokine Syndrome
KS: Kaposi Sarcoma
MCD: Multicentric Castleman Disease
MSM: men who have sex with men
mTOR: mammalian target of rapamycin
PEL: primary effusion lymphoma
PT: Post-transplant
R: recipient
SOT: Solid organ transplant
vIRFS: viral interferon regulatory factors
